# A Systematic Comparison of Age, Comorbidity and Frailty of Two Defined ICU Populations in the German Helios Hospital Group from 2016–2021

**DOI:** 10.3390/jcm14072332

**Published:** 2025-03-28

**Authors:** Kristina Hoffmann, Sven Hohenstein, Jörg Brederlau, Jan Hirsch, Heinrich V. Groesdonk, Andreas Bollmann, Ralf Kuhlen

**Affiliations:** 1 Institute for Social Medicine, Faculty of Medicine, Occupational Medicine and Public Health, Leipzig University, 04103 Leipzig, Germany; 2 Clinical Trial Management & Real World Data, Helios Health Institute, 13125 Berlin, Germany; 3 Department for Critical Care Medicine, Helios Clinic Berlin Buch, 13125 Berlin, Germany; 4 Department for Interdisciplinary Intensive Care Medicine and Intermediate Care, Helios Clinic Erfurt, 99089 Erfurt, Germany; 5 Real World Evidence and Health Technology Assessment, Helios Health Institute, 13125 Berlin, Germany; 6 Helios Health Institute, 13125 Berlin, Germany

**Keywords:** ICU patient characteristics, ICU patient demographics, trend analysis, age, frailty, comorbidity, COVID-19, ICU definition

## Abstract

**Background/Objectives:** The demographic change raises concerns about the provision of adequate, long-term healthcare. Our study was driven by the decision to test other studies’ findings about how patients’ age and comorbidities are significantly increasing in German intensive care units (ICUs) over time. The goal of this study was to analyze the age and age-related characteristics, e.g., comorbidities and frailty, in ICU populations from 86 hospitals in the German Helios Group over a period of 6 years. **Methods**: For this retrospective observational study, we derived two different definitions of ICU cases, with (i) CodeBased ICU cases being defined by typical ICU procedures (e.g., OPS 8-980, 8-98f and/or duration of ventilation > 0 h) derived from the German administrative dataset of claims data according to the German Hospital Remuneration Act and (ii) BedBased ICU cases being based on the actual presence of a patient on a designated ICU bed; this was taken from the Helios hospital bed classification system. For each ICU definition, the size of the respective ICU population, age, Elixhauser Comorbidity Index (ECI) and Hospital Frailty Risk Score (HFR) were analyzed. Further patient characteristics, treatments and outcomes are reported. Trends in cases with and without COVID-19 were analyzed separately. **Results**: We analyzed a total of 6,204,093 hospital cases, of which 281,537 met the criteria for the CodeBased ICU definition and 457,717 for the BedBased ICU definition. A key finding of our study is that a change in age in absolute and relative terms is observable and statistically significant: the mean age of CodeBased ICU cases, 68.7 (14.4/−0.06), is marginally decreasing, and that of BedBased ICU cases, 69.1 (15.9/0.07) (both with a *p*-value of <0.001), is marginally increasing. Age analysis excluding COVID-19 cases does not change this key finding. A longitudinal analysis shows a continuously decreasing number of ICU admissions and a marginally positive trend of patients who are 60–69 and ≥80 years old: CodeBased ICU, 1.04/1.02; BedBased ICU, 1.03/1.03, all with a *p*-value of <0.001. A severity analysis based on the ECI and HFS shows that both are higher in CodeBased ICU cases (2021 ECI:18.0 (12.9); HFS: 10.7 (7.3); both *p*-values < 0.001) than in BedBased ICU cases (2021 ECI: 12.3 (12.4); HFS: 7.4 (7.1); *p*-values of 0.3 and 0.12). Further testing results per definition are reported. **Conclusions**: The observed age-related trends suggest that there has been a further increase in demand for intensive care from a frailer population. We recommend further studies to critically evaluate the increasing frailty within the ICU population and to test the associated presumed need for increased ICU capacities.

## 1. Introduction

The world’s health systems are being challenged due to population aging [1]. Europe is facing a significant demographic change, with the median age increasing by 4.5 years between 2019 and 2050 and the old-age dependency ratio (the number of individuals aged ≥65 years per 100 people of working age, defined as those aged >20 to <64 years) projected to double [2,3]. Further, a rapid expansion in the number of people aged >85 years has raised concerns about the provision of adequate, long-term healthcare in Europe [4]. In 2019, Eastern Germany had one of the highest shares in the EU, with 28.9% (in Chemnitz) of its population aged ≥65 years. In 2050, up to 1/3 of the German population is expected to be aged >60 years [5]. Studies show a stagnation of the proportion of life in good health, which implies poor health conditions in the additional years [1]. A decrease in death rates since 1990 and an increase in age-standardized years lived with disability has been observed globally [6]. Disability as an outcome and frailty and co- or multimorbidity as risk factors are linked and are associated with an increase in physician visits and hospitalizations, especially in the elderly [7,8,9,10]. In studies, frailty has emerged as a syndrome characterized by criteria such as unintentional weight loss, self-reported exhaustion, weak grip strength, slow walking speed and physical activity. Therefore, it is a mostly underestimated risk factor for an often unrecognized and particularly vulnerable intensive care unit (ICU) population with significantly poorer outcomes, suggesting the integration of a frailty assessment prior to ICU admission [11,12,13]. Frailty is a characteristic of almost half of the elderly patients in ICUs in Europe and is a robust predictor of short survival, making it more meaningful than age as a singular variable [14,15]. The elderly and the chronically ill seem to be the key drivers for healthcare service demand and costs, including critical care, due to their disproportionately high medical needs [16]. In addition, there are methodological inconsistencies as to what is meant by an ICU [17,18,19,20]. The COVID-19 pandemic has made this methodological deficit clear and has highlighted various approaches to improve the use of existing resources [21,22].

Our study was driven by the decision to test other studies’ findings about how patients’ age and comorbidities are significantly projected to increase in German ICUs over time [23,24,25,26,27]. The goal of this study was to analyze the age and age-related characteristics, such as comorbidities and frailty, in the ICU populations from 86 hospitals in the German Helios Group over a period of 6 years. For this purpose, we used routine data-based definitions of ICU that we recently published [20]. The impact of COVID-19 on the characteristics of the ICU cohort was analyzed separately.

## 2. Materials and Methods

For this retrospective observational study, patients’ data were stored in a double-pseudonymized form. Data use was approved by the local ethics committee (File number/Aktenzeichen: 490/20-ek) and the Helios Kliniken GmbH data protection authority. Considering that this was a retrospective analysis of double-pseudonymized administrative data, individual informed consent was not obtained. We used a routine dataset that was described in detail in a recent publication [20]. Briefly, we used two different definitions of ICU cases, with (i) the CodeBased ICU definition being defined by ICU typical procedures (according to the German Classification System of Operations and Procedures (OPS) to code surgical interventions and medical procedure used in billing and documentation of treatments with the ICU Codes 8-980, 8-98f and/or duration of ventilation > 0 h) and (ii) the BedBased ICU definition being based on the actual presence of a patient on a designated ICU bed according to the Helios hospital bed classification system (Table 1).

We analyzed in-patient cases of patients ≥ 18 years of age who were admitted to an ICU in the period from 1 January 2016 to 31 December 2021. We chose this time period due to the adjustment of the pseudonymization of patient data beginning in 2016 (case and patient numbers are available in one system so that patients can be identified in several cases within a hospital), which makes comparisons with data from before 2016 difficult. We distinguished cases by definition according to all cases, cases without COVID-19 and only COVID-19 cases in absolute and relative numbers.

The in-hospital mortality rate was defined as the number of cases in which death was the reason for hospital discharge divided by all cases. We excluded cases that were transferred to another hospital or discharged for unspecified reasons. The length of hospital stay (LOSh; measured in nights) was defined as the number of nights spent in the hospital [28]. We excluded cases with a length of stay in an intensive care unit (LOSi; measured in days) with a duration equal to 0. Mechanical ventilation was defined as OPS 8-70x, 8-71x or a duration of ventilation of >0, measured in hours. Based on the patients’ comorbidities, we computed the Hospital Frailty Risk Score, which provides hospitals and health systems with a systematic method of screening for frailty to identify groups of patients who are at greater risk of adverse outcomes in order to adopt a frailty-attuned approach [29]. Technically, the score is a weighted sum of 109 comorbidities (defined as three-digit ICD codes). The authors distinguished three risk groups based on the score: low risk (score < 5), intermediate risk (score 5–15) and high risk (score > 15). The COVID-19 sub-cohort was defined as all cases with a SARS-CoV-2 infection using ICD-10-Code ICD U07.1.

For each ICU definition, we defined the size of the ICU population as the number of all cases meeting the respective criteria. We further compared the patient characteristics, clinical course and outcomes based on all cases with respect to age (reported in years of age per calendar year), sex, admission rates, Hospital Frailty Risk Score, Elixhauser Comorbidity Index, treatment episodes with extracorporeal membrane oxygenation (ECMO; 8-852.0/8-852.3/8-852.6), in-hospital mortality rate, in-hospital mortality rate of mechanically ventilated patients, length of stay in the hospital (LOSh) and length of stay in the ICU (LOSi) for each definition both with and without COVID-19.

For patient age we report results based on both numerical values and age groups. Since the length-of-stay variables (in a hospital or ICU) were positively skewed, we transformed them via an inverse hyperbolic sine in order to approximate normal distributions [30]. We report statistics for the Elixhauser Comorbidity Index (ECI) by using the Agency for Healthcare Research and Quality (AHRQ) algorithm, which can yield negative values for ECI (i.e., ECI < 0), and for the Hospital Frailty Risk Index based on both numerical values and risk groups [31,32]. Administrative data were extracted from QlikView (QlikTech, Radnor, PA, USA). Inferential statistics were generated in the R environment for statistical computing (version 4.0.2, 64-bit build) [33]. For all tests, we applied a two-tailed 5% error criterion for significance. For statistical tests of trends, we employed logistic regression for categorical variables and linear regression for numerical variables. The analysis of the length of stay (in a hospital or ICU) variable was performed via linear models. We report proportions, means, standard deviations and *p*-values. For the comparison of the proportions of selected treatments and outcomes in the different cohorts, we used logistic regression with a logit link function. We report proportions, odds ratios and *p*-values. Patients with missing information on discharge reason were excluded from in-hospital mortality analyses. For the years in which COVID-19 was considered (2020 and 2021), we tested for the robustness of possible trends by performing sensitivity and subgroup analyses. We calculated the age trends per year once with all cases and once without COVID-19 cases (excluding all cases with COVID-19). Due to the average of 1 million structured and complete data points (patient cases) that were collected monthly per survey year (6 years in total), valid retrospective trends could be determined [34,35]. Additional information on the procedure and the results of the sensitivity and subgroup analyses is provided in Appendix A.

## 3. Results

A total of 6,204,093 cases were analyzed in the 6 years of the study period. The development of the ICU case numbers for the CodeBased and BedBased ICU definitions is depicted in Table 2 for all cases, cases without COVID-19 and only COVID-19 cases.

The key finding of our study is that the distribution of patients across the respective age cohorts changed over time, and the total number of cases per year decreased for both definitions (Table 3).

**Figure 1:**
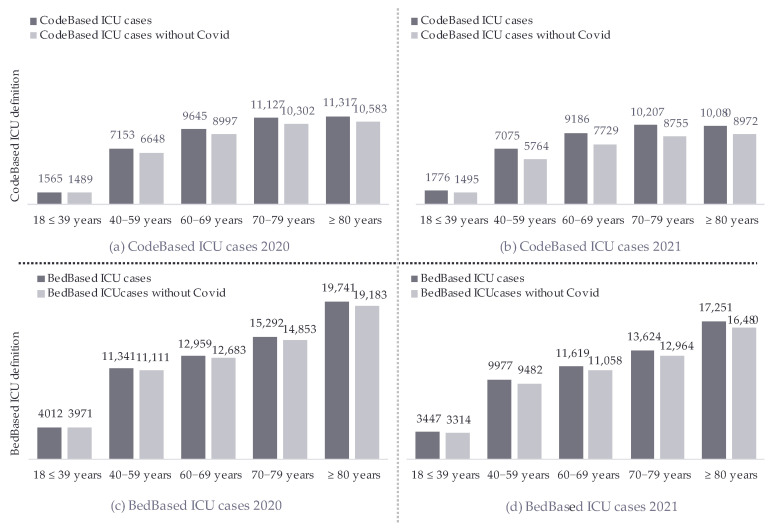
Age-group comparison of CodeBased ICU and BedBased ICU cases with and without COVID-19 in 2020 and 2021.

The mean age for the CodeBased ICU definition, 68.7 (14.4), slightly decreased, and that for the BedBased ICU definition, 69.1 (15.9) (both with a *p*-value of <0.001), slightly increased over the years. Despite the denominator difference in the definitions, the age cohort trends were comparable: patients aged 60–69 years and those aged ≥80 years marginally increased, while patients aged 40–59 years and 70–79 years marginally decreased over time. Additionally, there was an increase in relative values in the age cohorts of 60–69 and ≥80 years, while a decrease in the absolute numbers of admission was observed over the years. The sensitivity and subgroup analyses prove the robustness of the findings. The age analysis without COVID-19 cases did not change the key finding (Figure 1).

**Figure 2:**
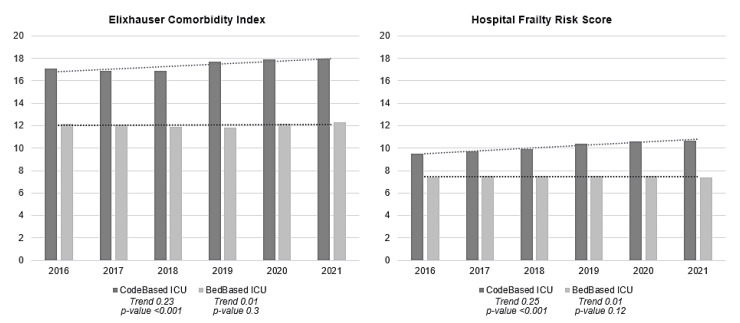
Comparison of CodeBased ICU and BedBased ICU cases in terms of the Elixhauser Comorbidity Index and Hospital Frailty Risk Score for 2016–2021 with the respective trend lines and with COVID-19 cases included.

The Elixhauser Comorbidity Index of CodeBased ICU cases was, on average, six points higher than that of BedBased ICU cases. The distribution of the score differed depending on the definition; when using the CodeBased ICU definition, 85% of the population had an ECI ≥5, and there was a marginally increasing trend of 1.05 (*p*-value < 0.001). According to the BedBased ICU definition, this proportion was 69%, with a constant trend of 1.00 (*p*-value: 0.066). On this basis, the BedBased ICU definition had higher proportions in the remaining ECI groups. The HFR in CodeBased ICU cases was approximately three points higher than that in BedBased ICU cases. Both were, on average, in the range of HFR intermediate risk (Figure 2 on the comparison of CodeBased ICU and BedBased ICU cases in terms of the ECI and HFR). The distribution of patients meeting the respective definitions differed as follows: most CodeBased ICU patients had intermediate risk, while BedBased ICU patients had low risk, both with a *p*-value of <0.001 (Table 4).

The comparison of the definitions shows significant differences, particularly in the absolute number of treatments, e.g., mechanical ventilation, in all cases in 2016 (CodeBased: 24,754 (43%) vs. BedBased: 10,990 (12%), both with a *p*-value of *p*-value < 0.001). In addition, these differences apply to the outcomes, e.g., the in-hospital mortality and in-hospital mortality of mechanically ventilated patients (Table 4).

## 4. Discussion

The goal of this study was to analyze the age and age-related characteristics, such as comorbidities and frailty, in the ICU populations from 86 hospitals in the German Helios Group over a period of 6 years. We found that, in absolute and relative terms, the change in age is observable and statistically significant but not as severe as expected. The high number of cases included in our study (6,204,093) and the significant trends observed support the robustness of our findings.

The longitudinal analysis shows a continuously decreasing absolute number of ICU admissions and a marginally positive trend of patients aged 60–69 and ≥80 years, which is associated with relevant health issues that reflect the changes in the healthcare needs of patients requiring ICU admission [36]. Therefore, the ICU population shows a relative increased share of older patients hereby comparing the proportion of these age groups to their proportion in the general population. To cover this changing demand, further factors such as limited workforce [37,38,39,40,41], workforce absenteeism and ICU bed allocation systematics [42,43,44,45], admission behavior as healthcare utilization and generally comparable ICU data [20,46,47], a fragmented and unstandardized data landscape [48,49,50] needs to be considered. Therefore, today, it is difficult to conduct a reliable “status quo” analysis as a valid basis for the prediction of ICU demand with an aging population [51,52]. A data-based comparative analysis of the development of the overall population, hospital populations and ICU populations can provide insights into future healthcare demands.

The severity analysis based on the Elixhauser Comorbidity Index (ECI) and Hospital Frailty Risk Score (HFR) shows that both are higher in the CodeBased ICU definition than in the BedBased ICU definition. This finding is consistent with many studies that have shown that not all patients who are actually in an ICU receive typical ICU treatments [20,53,54]. However, for both definitions, the ECI and HFR increase with age. A meta-analysis by Yan et al., 2022, confirms a significantly increased risk of peri-operative, prolonged LOS and mortality in fragile patients [55]. Hongye Zhao et al. show in their retrospective cohort study of 1164 patients that treatment with antibiotics also takes significantly longer and all-cause mortality is particularly high compared to robust patients of the same age [56]. Looking at the COVID-19 pandemic and focusing on spinal surgery, Dengler et al. found that there was an increase in high-fragility patients associated with comorbidities and a resulting reduction in surgery in this patient group [57]. Studies recommend the implementation of standardized frailty screening and the establishment of frailty clinical care pathways to better meet the needs of these patient groups [58,59,60].

Additionally, the observed differences are due to the definitions themselves. The CodeBased ICU definition is comparatively restrictive, as it considers only the OPS and/or length of stay. However, the BedBased ICU definition could be too liberal, as it is unclear who uses IMC beds. The number of patients with an ECI of <5 might be an indicator. The difference in the definitions based on an ECI of ≥5 is reasonable because the CodeBased ICU definition excludes all that is not OPS, so the population according to the CodeBased ICU definition is, in total, smaller, but those that are included are most often found in the group with an ECI of ≥5. An overestimation of the actual ICU population and its distribution of comorbidities is likely with the CodeBased ICU definition. Therefore, the BedBased ICU definition seems more plausible. This also applies to the observation for the HFR; the consideration of comorbidities leads to an overestimation of the population according to the CodeBased ICU definition. The finding of a frailer population in ICUs and IMCs is conclusive and corresponds to the trend for the age group of ≥80 years, which is represented in the BedBased ICU definition. In summary, the challenge is determining what to consider when planning ICU capacities. With the BedBased ICU definition, there are more patients but with less intensive care effort, and the opposite is true with the CodeBased ICU definition, though comorbidities and fragility increase with both.

The results of this study provide insights into care practices beyond the scope of this study that we would like to address. Testing the CodeBased and BedBased ICU definitions to determine which ICU population they cover reveals clear differences that are of particular relevance for capacity planning and management. In particular, the result of the mechanically ventilated cases stands out. Due to the different inclusion criteria of the definitions (the CodeBased ICU definition is restrictive due to its hard criteria, such as OPS, whereas the BedBased ICU definition is moderate because it refers to bed occupancy, with a focus on patients with a stay in an ICU or IMC (Table 1), independent of ICU procedures), the BedBased definition underestimates the number of mechanically ventilated patients. This is an important finding for further studies, as it implies that another definition that includes, for example, all cases in an ICU (without IMCs) should be tested. The insights into the increase in mechanically ventilated cases, e.g., from 2018 to 2019, in the CodeBased population from 47% to 57% raises questions about coding practices, updates in OPS codes or referral practices that would benefit from a deeper analysis. The age distributions remain unaffected by these results, as the comparison of the age cohorts across both definitions clearly shows (Table 3).

Finally, our finding of an increasing trend in the proportion of older patients in German ICUs is consistent with international studies, which also have documented similar trends in different healthcare settings, e.g., Australia/New Zealand, the USA, Italy, Russia and India [61,62,63,64,65,66].

Our study has several methodological limitations.

(1) Due to the changes in pseudonymization from 2016 onwards, the data prior to this are no longer comparable; thus, the analysis was limited to the 6-year study period. This limitation may prevent important trends prior to this point in time from being recorded. Nevertheless, we conclude that this observation period is sufficient to show significant age trends because we used our total population of 6,204,093 cases as the basis. The measurement interval was based on the patient’s individual, daily admission date, which we included in the study as a mean value per year; this is considered consistent and in accordance with best practices using the analytical methods described. Furthermore, a longer observation period (>2 years) of the age distribution of cases with and without COVID-19 would show the longitudinal trend and effect of COVID-19 more clearly.
(2) The CodeBased and BedBased ICU definitions have limitations. In the BedBased ICU definition, bed occupancy includes patients who may not have a hard ICU indication. The CodeBased ICU definition reflects only part of the reality because it includes patients with intensive medical therapy but excludes those with intensive monitoring (as is common in IMCs).
(3) The selected definitions are not clearly replicable and controllable. The list of approaches is not necessarily finite. Due to the lack of an ICU definition, the CodeBased ICU definition was adopted as the first-quality definition, and the BedBased ICU definition was taken from the Helios Hospital Group’s own bed classification. According to this, hospitals are required to provide information on their completed services; here, there are challenges in coming to the same understanding of what is meant by “ICU”—disparities are to be expected [67].
(4) The weighting of age as a determining variable within the HFS is not definitive. The use of the two scores is based on administrative data and may be influenced by coding practices. The accuracy of HFS is being discussed in the scientific community, and attempts are being made to optimize it [68,69].
(5) At present, there is no uniform (inter)national definition of what is meant by “ICU” or “IMC”. The introduction of a uniform national ICU definition is the responsibility of legislators. Initial steps can be taken at the level of individual hospitals, but only a legal requirement can create a common definition. The introduction of a uniform definition of ICUs with clear criteria will not replace or override clinical assessments or the individual needs of patients.
(6) Additionally, a differentiated approach should be used to assess the admission behavior, therapeutic value, and quality of outcomes for elderly patients in ICUs, acknowledging the challenges in balancing patient-centered care with ICU admission, such as discharge regulations and coding behavior.

## 5. Conclusions

The goal of this study was to analyze the age and age-related characteristics, such as comorbidities and frailty, in the ICU populations from 86 hospitals in the German Helios Group over a period of 6 years. We found that the age groups of 60–69 and ≥80 years exhibit a minor positive trend, with the total number of hospitalized cases exhibiting a stable downward trend. Depending on the segment of ICU population (ICU definition) the severity of illness of ICU patients in terms of ECI and HFR is increasing. However, for both definitions under analysis, the ECI and HFR increase with age. The relevance of these findings on healthcare indicates that there is an increasing demand for intensive care from a frailer population.

We recommend further studies to critically evaluate the increasing frailty within the ICU population and to test the associated presumed need for increased ICU capacities. It is essential to consider the global shortage of healthcare workforces and the likely absence of an improvement in the quality of life for frail patients. Additionally, a differentiated approach should be used to assess the admission behavior, therapeutic value, and quality of outcomes for elderly patients in ICUs, acknowledging the challenges in balancing patient-centered care with ICU admission, such as discharge regulations and coding behavior.

Further, we recommend a data-based definition of intensive care capacity planning, with clear admission and discharge criteria for ICUs and IMCs. Continuous real-world studies should help guide future development and enable potential adjustments as quickly as possible.

## Figures and Tables

**Table 1:** Derived ICU definitions.

Nr.	Abbreviation	Definition and Its Criteria	Reason for Choice	Total Number of All Cases per Definition (*n*) and Proportion of All Cases (%)
1	CodeBased ICU	Based on the ICU definition of the German Initiative for Quality in Medicine OPS 8-980, 8-98f and/or Duration of ventilation > 0 h	Reference definition Is used in numerous publications Contains two robust indicators for intensive care treatment Relevant to hospital revenue	281,537 (4.5%)
2	BedBased ICU	Based on the bed classification system of the German Helios Hospital Group. It includes a combination of the Hospital Section Intensive Care Unit and Intermediate Care Unit combined Hospital bed classified as an ICU bed; Hospital bed classified as an IMC bed	Indicates intensive care treatment Provides an overview of each ICU designation and the nursing location Most plausible approximation of real-world ICU populations [20]	457,717 (7.4%)

**Table 2:** Development of the case numbers over the years and according to the definition of in-patient cases.

	All Cases *n* (%)	Cases per Definition *n* (%)		Cases without COVID-19 *n* (%)		Only COVID-19 Cases *n* (%)	
Definition	None	CodeBased ICU	BedBased ICU	CodeBased ICU	BedBased ICU	CodeBased ICU	BedBased ICU
Year/Total *n* Cases	6,204,093	281,537	457,717	273,140	453,553	8397	4164
2016	1,068,610 (17,2%)	57,414 (20%)	88,317 (19%)	57,414 (21%)	88,317 (19%)	0 (0%)	0 (0%)
2017	1,076,906 (17,4%)	54,319 (19%)	91,902 (20%)	54,319 (20%)	91,902 (20%)	0 (0%)	0 (0%)
2018	1,071,445 (17,3%)	50,244 (18%)	81,822 (18%)	50,244 (18%)	81,822 (18%)	0 (0%)	0 (0%)
2019	1,073,693 (17,3%)	40,429 (14%)	76,413 (17%)	40,429 (15%)	76,413 (17%)	0 (0%)	0 (0%)
2020	963,883 (15,5%)	40,807 (14%)	63,345 (14%)	38,019 (14%)	61,801 (14%)	2788 (33%)	1544 (37%)
2021	949,556 (15,3%)	38,324 (14%)	55,918 (12%)	32,715 (12%)	53,298 (12%)	5609 (67%)	2620 (63%)

**Table 3:** Age group distribution of CodeBased ICU and BedBased ICU cases by year.

CodeBased ICU: All Cases—Includes the Impact of COVID-19									
Age Groups/Year	2016	2017	2018	2019	2020	2021	Trend (a)	Trend (*n*)	*p* Value
18–39 years	2416 (4.2%)	2238 (4.1%)	2028 (4.0%)	1721 (4.3%)	1565 (3.8%)	1776 (4.6%)	→	1.01	0.12
40−59 years	10,733 (19%)	9745 (18%)	9015 (18%)	7166 (18%)	7153 (18%)	7075 (18%)	↓	0.99	0.025
60−69 years	11,882 (21%)	11,694 (22%)	11,121 (22%)	9412 (23%)	9645 (24%)	9186 (24%)	↑	1.04	<0.001
70−79 years	17,770 (31%)	16,551 (30%)	14,850 (30%)	11,355 (28%)	11,127 (27%)	10,207 (27%)	↓	0.96	<0.001
≥80 years	14,613 (25%)	14,091 (26%)	13,230 (26%)	10,775 (27%)	11,317 (28%)	10,080 (26%)	↑	1.02	<0.001
Mean Age	69.3 (14.4)	69.5 (14.3)	69.5 (14.2)	69.4 (14.2)	69.5 (14.1)	68.7 (14.4)	↓	−0.06	<0.001
Total Cases	57,414	54,319	50,244	40,429	40,807	38,324	↓	x	x
**BedBased ICU: All Cases—Includes The Impact of COVID-19**									
**Age Groups/Year**	**2016**	**2017**	**2018**	**2019**	**2020**	**2021**	**Trend (a)**	**Trend (*n*)**	***p* value**
18–39 years	5588 (6.3%)	5844 (6.4%)	5314 (6.5%)	4874 (6.4%)	4012 (6.3%)	3447 (6.2%)	→	1.00	0.3
40−59 years	16,695 (19%)	16,920 (18%)	14,810 (18%)	13,752 (18%)	11,341 (18%)	9977 (18%)	↓	0.99	<0.001
60−69 years	16,520 (19%)	17,428 (19%)	15,888 (19%)	15,340 (20%)	12,959 (20%)	11,619 (21%)	↑	1.03	<0.001
70−79 years	24,984 (28%)	25,425 (28%)	21,856 (27%)	19,502 (26%)	15,292 (24%)	13,624 (24%)	↓	0.95	<0.001
≥80 years	24,530 (28%)	26,285 (29%)	23,954 (29%)	22,945 (30%)	19,741 (31%)	17,251 (31%)	↑	1.03	<0.001
Mean Age	68.7 (16.0)	68.9 (16.1)	69.0 (16.1)	69.0 (16.0)	69.1 (16.0)	69.1 (15.9)	↑	0.07	<0.001
Total Cases	88,317	91,902	81,822	76,413	63,345	55,918	↓	x	x

→ = trend remains unchanged, stable; ↓ = trend is decreasing; ↑ = trend is increasing.

**Table 4:** Patient characteristics, clinical course and outcomes for the CodeBased and BedBased ICU definitions.

Sex/Female Proportion		2016	2017	2018	2019	2020	2021	Trend	*p* Value
CodeBased	All cases	23,740 (41%)	22,375 (41%)	20,794 (41%)	16,509 (41%)	16,422 (40%)	15,232 (40%)	↓	<0.001
	Cases without COVID-19	23,740 (41%)	22,375 (41%)	20,794 (41%)	16,509 (41%)	15,433 (41%)	13,202 (40%)	↓	<0.001
BedBased	All cases	40,039 (45%)	41,584 (45%)	36,917 (45%)	34,180 (45%)	28,185 (44%)	24,986 (45%)	↓	<0.001
	Cases without COVID-19	40,039 (45%)	41,584 (45%)	36,917 (45%)	34,180 (45%)	27,541 (45%)	23,948 (45%)	↓	0.002
**Elixhauser Comorbidity Index (ECI)**		**2016**	**2017**	**2018**	**2019**	**2020**	**2021**	**Trend**	***p* value**
CodeBased	All cases	17.1 (12.9)	16.9 (12.8)	16.9 (12.6)	17.7 (12.8)	17.9 (13.0)	18.0 (12.9)	↑	<0.001
	ECI < 0	4506 (7.8%)	4104 (7.6%)	3619 (7.2%)	2730 (6.8%)	2862 (7.0%)	2600 (6.8%)	↓	<0.001
	ECI 0	2512 (4.4%)	2448 (4.5%)	2157 (4.3%)	1541 (3.8%)	1558 (3.8%)	1470 (3.8%)	↓	<0.001
	ECI 1−4	3278 (5.7%)	3206 (5.9%)	2976 (5.9%)	2194 (5.4%)	2034 (5.0%)	1811 (4.7%)	↓	<0.001
	ECI ≥ 5	47,118 (82%)	44,561 (82%)	41,492 (83%)	33,964 (84%)	34,353 (84%)	32,443 (85%)	↑	<0.001
	Cases without COVID-19	17.1 (12.9)	16.9 (12.8)	16.9 (12.6)	17.7 (12.8)	18.0 (13.0)	18.3 (13.0)	↑	<0.001
BedBased	All cases	12.2 (12.5)	12.1 (12.3)	11.9 (12.2)	11.8 (12.2)	12.2 (12.3)	12.3 (12.4)	→	0.3
	ECI < 0	14,280 (16%)	14,311 (16%)	12,607 (15%)	12,182 (16%)	9734 (15%)	8269 (15%)	↓	<0.001
	ECI 0	7700 (8.7%)	8235 (9.0%)	7390 (9.0%)	6957 (9.1%)	5808 (9.2%)	5189 (9.3%)	↑	<0.001
	ECI 1−4	6573 (7.4%)	6813 (7.4%)	6318 (7.7%)	6010 (7.9%)	4721 (7.5%)	4121 (7.4%)	→	0.7
	ECI ≥ 5	59,764 (68%)	62,543 (68%)	55,507 (68%)	51,264 (67%)	43,082 (68%)	38,339 (69%)	→	0.066
	Cases without COVID-19	12.2 (12.5)	12.1 (12.3)	11.9 (12.2)	11.8 (12.2)	12.0 (12.3)	12.2 (12.4)	→	0.10
**Hospital Frailty Risk Score (HFR)**		**2016**	**2017**	**2018**	**2019**	**2020**	**2021**	**Trend**	***p* value**
CodeBased	All cases	9.5 (7.4)	9.7 (7.5)	9.9 (7.4)	10.4 (7.3)	10.6 (7.4)	10.7 (7.3)	↑	<0.001
	HFR < 5	19,084 (33%)	17,465 (32%)	15,380 (31%)	10,846 (27%)	10,526 (26%)	9488 (25%)	↓	<0.001
	HFR 5−15	25,715 (45%)	24,536 (45%)	23,165 (46%)	19,493 (48%)	19,998 (49%)	19,120 (50%)	↑	<0.001
	HFR > 15	12,615 (22%)	12,318 (23%)	11,699 (23%)	10,090 (25%)	10,283 (25%)	9716 (25%)	↑	<0.001
	Cases without COVID-19	9.5 (7.4)	9.7 (7.5)	9.9 (7.4)	10.4 (7.3)	10.5 (7.4)	10.6 (7.3)	↑	<0.001
BedBased	All cases	7.4 (7.2)	7.5 (7.2)	7.5 (7.2)	7.5 (7.1)	7.5 (7.2)	7.4 (7.1)	→	0.12
	HFR < 5	42,716 (48%)	44,167 (48%)	38,893 (48%)	36,238 (47%)	29,870 (47%)	26,726 (48%)	↓	<0.001
	HFR 5−15	31,953 (36%)	33,458 (36%)	30,223 (37%)	28,250 (37%)	23,601 (37%)	20,748 (37%)	↑	<0.001
	HFR > 15	13,648 (15%)	14,277 (16%)	12,706 (16%)	11,925 (16%)	9874 (16%)	8444 (15%)	→	0.3
	Cases without COVID-19	7.4 (7.2)	7.5 (7.2)	7.5 (7.2)	7.5 (7.1)	7.4 (7.2)	7.2 (7.0)	↓	0.001
**Mechanical ventilation (MV)**		**2016**	**2017**	**2018**	**2019**	**2020**	**2021**	**Trend**	***p* value**
CodeBased	All cases	24,754 (43%)	24,320 (45%)	23,810 (47%)	22,939 (57%)	23,284 (57%)	23,444 (61%)	↑	<0.001
	Cases without COVID-19	24,754 (43%)	24,320 (45%)	23,810 (47%)	22,939 (57%)	20,999 (55%)	18,563 (57%)	↑	<0.001
BedBased	All cases	10,990 (12%)	12,257 (13%)	11,309 (14%)	10,342 (14%)	8827 (14%)	8126 (15%)	↑	<0.001
	Cases without COVID-19	10,990 (12%)	12,257 (13%)	11,309 (14%)	10,342 (14%)	8058 (13%)	6618 (12%)	→	0.8
**In-hospital mortality**		**2016**	**2017**	**2018**	**2019**	**2020**	**2021**	**Trend**	***p* value**
CodeBased	All cases	9802 (20%)	9594 (21%)	9321 (22%)	8461 (25%)	9110 (26%)	9648 (30%)	↑	<0.001
	Cases without COVID-19	9802 (20%)	9594 (21%)	9321 (22%)	8461 (25%)	7953 (25%)	7390 (27%)	↑	<0.001
BedBased	All cases	6824 (8.5%)	7371 (8.9%)	6600 (9.0%)	5851 (8.5%)	5343 (9.3%)	4889 (9.8%)	↑	<0.001
	Cases without COVID-19	6824 (8.5%)	7371 (8.9%)	6600 (9.0%)	5851 (8.5%)	4867 (8.7%)	4145 (8.7%)	→	0.8
**In-hospital mortality of MV patients**		**2016**	**2017**	**2018**	**2019**	**2020**	**2021**	**Trend**	***p* value**
CodeBased	All cases	7975 (39%)	7943 (40%)	7813 (40%)	7337 (39%)	7867 (41%)	8458 (44%)	↑	<0.001
	Cases without COVID-19	7975 (39%)	7943 (40%)	7813 (40%)	7337 (39%)	6801 (40%)	6324 (42%)	↑	0.007
BedBased	All cases	3513 (38%)	3787 (37%)	3310 (35%)	2908 (34%)	2636 (36%)	2527 (38%)	↓	0.018
	Cases without COVID-19	3513 (38%)	3787 (37%)	3310 (35%)	2908 (34%)	2300 (34%)	1929 (35%)	↓	<0.001
**Length of stay in hospital (LOSh)**		**2016**	**2017**	**2018**	**2019**	**2020**	**2021**	**Trend**	***p* value**
CodeBased	All cases	15.6 (15.6); 11.0 [6.0–20.0]	15.4 (15.8); 11.0 [6.0–20.0]	15.1 (15.9); 11.0 [6.0–19.0]	16.0 (16.7); 11.0 [6.0–20.0]	15.5 (16.3); 11.0 [6.0–20.0]	15.9 (16.4); 11.0 [6.0–20.0]	↓	<0.001
	Cases without COVID-19	15.6 (15.6); 11.0 [6.0–20.0]	15.4 (15.8); 11.0 [6.0–20.0]	15.1 (15.9); 11.0 [6.0–19.0]	16.0 (16.7); 11.0 [6.0–20.0]	15.0 (15.8); 11.0 [6.0–19.0]	15.2 (16.2); 11.0 [6.0–19.0]	↓	<0.001
BedBased	All cases	11.1 (11.2); 8.0 [4.0–14.0]	11.1 (11.1); 8.0 [4.0–14.0]	10.8 (11.5); 8.0 [4.0–14.0]	10.9 (11.7); 7.0 [4.0–14.0]	10.3 (11.3); 7.0 [4.0–13.0]	10.5 (11.6); 7.0 [4.0–13.0]	↓	<0.001
	Cases without COVID-19	11.1 (11.2); 8.0 [4.0–14.0]	11.1 (11.1); 8.0 [4.0–14.0]	10.8 (11.5); 8.0 [4.0–14.0]	10.9 (11.7); 7.0 [4.0–14.0]	10.0 (10.9); 7.0 [4.0–13.0]	10.1 (11.1); 7.0 [4.0–13.0]	↓	<0.001
**Length of stay in ICU (LOSi)**		**2016**	**2017**	**2018**	**2019**	**2020**	**2021**	**Trend**	***p* value**
CodeBased	All cases	7.0 (10.3); 4.0 [2.0–7.0]	7.0 (10.8); 4.0 [2.0–7.0]	6.8 (10.3); 3.0 [2.0–7.0]	7.6 (10.8); 4.0 [2.0–8.0]	7.8 (11.1); 4.0 [2.0–9.0]	8.3 (11.7); 4.0 [2.0–10.0]	↑	<0.001
	Cases without COVID-19	7.0 (10.3); 4.0 [2.0–7.0]	7.0 (10.8); 4.0 [2.0–7.0]	6.8 (10.3); 3.0 [2.0–7.0]	7.6 (10.8); 4.0 [2.0–8.0]	7.4 (10.7); 4.0 [2.0–8.0]	7.5 (11.2); 4.0 [2.0–8.0]	↑	<0.001
BedBased	All cases	3.5 (6.1); 2.0 [1.0–3.0]	3.5 (6.2); 2.0 [1.0–3.0]	3.4 (6.4); 2.0 [1.0–3.0]	3.5 (6.5); 2.0 [1.0–3.0]	3.5 (6.6); 2.0 [1.0–3.0]	3.6 (7.0); 2.0 [1.0–3.0]	↓	0.018
	Cases without COVID-19	3.5 (6.1); 2.0 [1.0–3.0]	3.5 (6.2); 2.0 [1.0–3.0]	3.4 (6.4); 2.0 [1.0–3.0]	3.5 (6.5); 2.0 [1.0–3.0]	3.4 (6.2); 2.0 [1.0–3.0]	3.3 (6.5); 2.0 [1.0–3.0]	↓	<0.001

→ = trend remains unchanged, stable; ↓ = trend is decreasing; ↑ = trend is increasing.

## Data Availability

The data presented in this study are available from the corresponding author upon request due to legal reasons.

## Appendix A

### Appendix A.1

Our initial rationale for including the pandemic years in our analysis was based on our findings that age trends did not appear to be altered during the pandemic compared to pre-pandemic years. However, we acknowledge that this may not fully account for the complex and multifaceted effects of COVID-19 on healthcare systems and patient behavior due to the disruptive effect of the COVID-19 pandemic. This is why we performed a Sensitivity Analysis and a Subgroup Analysis where we excluded the pandemic year (2020 and 2021) from our trend analysis (Table A1). This allowed us to compare the age trend observed during the normal period with those during the pandemic and this way asses the robustness of our findings. In doing so, we address a possible bias in age trends. We displayed these results in Figure 1 due to the stable trends in both cohorts.

**Table A1:** Comparison of age groups with all cases and all cases without COVID-19.

CodeBased ICU All Cases									
Age Groups/Year	2016	2017	2018	2019	2020	2021	Trend (a)	Trend (*n*)	*p* Value
18–39 years	2416 (4.2%)	2238 (4.1%)	2028 (4.0%)	1721 (4.3%)	1565 (3.8%)	1776 (4.6%)	→	1.01	0.12
40−59 years	10,733 (19%)	9745 (18%)	9015 (18%)	7166 (18%)	7153 (18%)	7075 (18%)	↓	0.99	0.025
60−69 years	11,882 (21%)	11,694 (22%)	11,121 (22%)	9412 (23%)	9645 (24%)	9186 (24%)	↑	1.04	<0.001
70−79 years	17,770 (31%)	16,551 (30%)	14,850 (30%)	11,355 (28%)	11,127 (27%)	10,207 (27%)	↓	0.96	<0.001
≥80 years	14,613 (25%)	14,091 (26%)	13,230 (26%)	10,775 (27%)	11,317 (28%)	10,080 (26%)	↑	1.02	<0.001
Mean Age	69.3 (14.4)	69.5 (14.3)	69.5 (14.2)	69.4 (14.2)	69.5 (14.1)	68.7 (14.4)	↓	−0.06	<0.001
Total Cases	57,414	54,319	50,244	40,429	40,807	38,324	↓	x	x
**CodeBased ICU All Cases Without COVID-19**									
**Age Groups/Year**	**2016**	**2017**	**2018**	**2019**	**2020**	**2021**	**Trend (a)**	**Trend (*n*)**	***p* Value**
18–39 years	2416 (4.2%)	2238 (4.1%)	2028 (4.0%)	1721 (4.3%)	1489 (3.9%)	1495 (4.6%)	→	1.01	0.2
40−59 years	10,733 (19%)	9745 (18%)	9015 (18%)	7166 (18%)	6648 (17%)	5764 (18%)	↓	0.99	<0.001
60−69 years	11,882 (21%)	11,694 (22%)	11,121 (22%)	9412 (23%)	8997 (24%)	7729 (24%)	↑	1.04	<0.001
70−79 years	17,770 (31%)	16,551 (30%)	14,850 (30%)	11,355 (28%)	10,302 (27%)	8755 (27%)	↓	0.96	<0.001
≥80 years	14,613 (25%)	14,091 (26%)	13,230 (26%)	10,775 (27%)	10,583 (28%)	8972 (27%)	↑	1.02	<0.001
Mean Age	69.3 (14.4)	69.5 (14.3)	69.5 (14.2)	69.4 (14.2)	69.5 (14.1)	69.1 (14.4)	↓	−0.01	0.7
Total Cases	57,414	54,319	50,244	40,429	38,019	32,715	↓	x	x
**BedBased ICU All Cases**									
**Age Groups/Year**	**2016**	**2017**	**2018**	**2019**	**2020**	**2021**	**Trend (a)**	**Trend (*n*)**	***p* Value**
18–39 years	5588 (6.3%)	5844 (6.4%)	5314 (6.5%)	4874 (6.4%)	4012 (6.3%)	3447 (6.2%)	→	1.00	0.3
40−59 years	16,695 (19%)	16,920 (18%)	14,810 (18%)	13,752 (18%)	11,341 (18%)	9977 (18%)	↓	0.99	<0.001
60−69 years	16,520 (19%)	17,428 (19%)	15,888 (19%)	15,340 (20%)	12,959 (20%)	11,619 (21%)	↑	1.03	<0.001
70−79 years	24,984 (28%)	25,425 (28%)	21,856 (27%)	19,502 (26%)	15,292 (24%)	13,624 (24%)	↓	0.95	<0.001
≥80 years	24,530 (28%)	26,285 (29%)	23,954 (29%)	22,945 (30%)	19,741 (31%)	17,251 (31%)	↑	1.03	<0.001
Mean Age	68.7 (16.0)	68.9 (16.1)	69.0 (16.1)	69.0 (16.0)	69.1 (16.0)	69.1 (15.9)	↑	0.07	<0.001
Total Cases	88,317	91,902	81,822	76,413	63,345	55,918	↓	x	x
**BedBased ICU All Cases Without COVID-19**									
**Age Groups/Year**	**2016**	**2017**	**2018**	**2019**	**2020**	**2021**	**Trend (a)**	**Trend (*n*)**	***p* Value**
18–39 years	5588 (6.3%)	5844 (6.4%)	5314 (6.5%)	4874 (6.4%)	3971 (6.4%)	3314 (6.2%)	→	1.00	0.8
40−59 years	16,695 (19%)	16,920 (18%)	14,810 (18%)	13,752 (18%)	11,111 (18%)	9482 (18%)	↓	0.99	<0.001
60−69 years	16,520 (19%)	17,428 (19%)	15,888 (19%)	15,340 (20%)	12,683 (21%)	11,058 (21%)	↑	1.03	<0.001
70−79 years	24,984 (28%)	25,425 (28%)	21,856 (27%)	19,502 (26%)	14,853 (24%)	12,964 (24%)	↓	0.95	<0.001
≥80 years	24,530 (28%)	26,285 (29%)	23,954 (29%)	22,945 (30%)	19,183 (31%)	16,480 (31%)	↑	1.03	<0.001
Mean Age	68.7 (16.0)	68.9 (16.1)	69.0 (16.1)	69.0 (16.0)	69.0 (16.1)	69.1 (15.9)	↑	0.07	<0.001
Total Cases	88,317	91,902	81,822	76,413	61,801	53,298	↓	x	x

→ = trend remains unchanged, stable; ↓ = trend is decreasing;↑ = trend is increasing.
